# Assessment and development of physical education teachers’ physical literacy: A systematic review

**DOI:** 10.1371/journal.pone.0307505

**Published:** 2024-07-18

**Authors:** Hang Yin, Roxana Dev Omar Dev, Kim Geok Soh, Fangyi Li, Menglong Lian

**Affiliations:** 1 Department of Sports Studies, Faculty of Educational Studies, Universiti Putra Malaysia, Serdang, Malaysia; 2 Zhengzhou Vocational College of Automobile Engineering, Zhengzhou, Henan Province, China; Southwest University, CHINA

## Abstract

In recent years, physical literacy (PL) has gained a great deal of attention in global academia. Children’s physical activity (PA) participation is severely underrepresented today, and students’ participation in PA and PL level development is strongly dependent on the PL levels of PE teachers. This study aims to offer information for PE teachers to improve their PL levels and for the future development of tools to assess the PL of PE teachers through a systematic review of studies assessing PL of PE teachers. The Preferred Reporting Items for Systematic Reviews and Meta-Analysis (PRISMA) was used to conduct a comprehensive and systematic search in six databases—Web of Science, Scopus, ScienceDirect, PubMed, ProQuest; and SportDiscus, and a total of 671 papers were retrieved, but after removing duplicates, article identification, and screening only eight papers met the inclusion criteria. This study’s results indicate a paucity of research related to PL among PE teachers, focusing on children, students, older adults, and children with disabilities. PE teachers performed poorly in the physical competence domain and better in the cognitive and affective domains, with a moderate level of overall PL. Only one instrument is currently available to assess PE teachers’ (perceived) PL, and other studies have used instrument components. Therefore, it was concluded that the current PE teachers’ PL level is not high. Also, because the concept of PL among PE teachers has not been standardized, no tool has been developed to evaluate the PL of PE teachers comprehensively and systematically. The CPD (continuing professional development) is considered an effective means of enhancing PL among PE teachers, and research should prioritize the development of CPD programs and tools that are specifically tailored to assess PL among PE teachers in the future.

## 1 Background

During childhood, there are considerable benefits to be gained from appropriate physical activity (PA), such as promoting physical growth and development, improving academic performance, sharpening willpower, and developing interpersonal communication skills [1–3]. However, in many countries, less than 40% of children are able to achieve the level of PA needed to maintain optimal health daily [4], and in some studies, this figure reaches a staggering 20% [5, 6], which means that worldwide, 8 out of 10 children are physically inactive. Physical inactivity has become one of the most critical factors contributing to life-threatening non-communicable diseases globally [7]. Inadequate PA is not only a serious threat to physical and mental health (e.g., obesity, hypertension, depression, etc.) but also a severe economic cost. For example, physical inactivity costs Australia $13 billion annually in financial losses, including medical expenses, loss of workforce, and premature death [8]. Although PA generally shows a downward trend throughout an individual’s entire life journey, if good and sufficient PA is carried out and maintained during childhood, it can delay the time when they become inactive [9] and prevent the development of harmful habits in the future, such as smoking, etc. [10]. Therefore, there is a growing call for children to become physically active, and it is urgent to make every effort to encourage children to participate in and maintain lifelong PA.

Physical literacy (PL), developed by Whitehead, is currently an effective underlying theory for encouraging lifelong PA participation [11, 12]. Since the concept was first introduced, it has attracted widespread scholarly attention worldwide [13]. The concept of PL has always been controversial and not unified; multiple scholars have attempted to define it [14–17]. However, the concept defined by Whitehead is widely accepted by the global academic community [17]. Whitehead defined physical literacy as "the motivation, confidence, physical competence, knowledge and understanding to maintain physical activity throughout the life course" [18]. The concept encompasses the affective, cognitive, and physical domains and is based on three main philosophical ideas: existentialism, monism, and phenomenology. These philosophical ideas are intertwined and reveal the true nature of PL by documenting an individual’s PA as it interacts with the world [19, 20].

Scholars and educational institutions around the world have recognized that PL is just as crucial as the educational value of literacy and numeracy [21, 22]. Meanwhile, Whitehead described the relationship between PL and PE, stating that PL is the ultimate goal of PE, and PE is the main means to achieve PL [23]. The concept of PL provides direction for active learning by students, active teaching by PE teachers, and active support and PE curriculum development by schools and governments. Therefore, PL is now integrated into physical education (PE) policies worldwide. For example, UNESCO’s QPE (Quality Physical Education) guidelines for PE policymakers use PL as a conceptual basis [24]. Subsequently, many countries or regions responded by setting PL as the target of PE. For example, SHAPE (Society of Health and Physical Educators) America’s third edition of the National Standards for K-12 Physical Education uses the term "physical literacy" instead of "physically educated" [25]. In the Chinese mainland, the government issued a school PE policy in 2016 that explicitly emphasizes the need to improve students’ PL [26]. In Hong Kong and Taiwan, PL was also introduced in the relevant policies [27]. The Australian Sports Commission has also developed PL standards applicable to their country [8]. Some countries in Europe are also working to integrate PL and PE. For example, PL has been included in the new 2021 curriculum for all ages in Greece, this concept has also been adopted in school sports in Denmark [28]. The majority of children’s time is devoted to being in school, so school is seen as the most essential and critical place for developing children’s and youths’ physical and mental health [29]. Adequate sports facilities, a positive PA climate, and a supportive school PA policy all have an impact on students’ active participation in PA and enhance their PL [30–32]. The most critical link, however, is the PE teacher. School PE policies need PE teachers to respond, PE classrooms need PE teachers to lead, and extracurricular PA needs PE teachers to guide. Everything that creates a connection with PA and PE activities within the school is inseparable from PE teachers. Meanwhile, PE teachers have a unique role because, when students are in school, they can have a direct positive impact on students’ physical learning and PA [33], which other subject teachers cannot do. Most importantly, the ultimate goal of PE is PL [34]. To pass on the various outcomes contained in PL to students and enable them to have PL, it is necessary to require PE teachers to understand PL, have PL as well as implement PL in PE [18]. In summary, promoting PL among PE teachers can be recognized as a key opportunity to bring significant health benefits to students [23]. However, many current studies have focused on children, children with disabilities, and older adults. This situation makes it particularly important to review the PL levels of PE teachers and to assess and develop PL among them.

This study aims to: first, systematically review the literature involving PE teachers’ PL levels and analyze current PE teachers’ PL levels and strategies for improving them. Second, find and critically evaluate assessment tools that have been used to assess PL levels of PE teachers in the current studies and to offer appropriate recommendations for future development of relevant tools.

## 2 Methods

This review strictly follows the reporting checklist of the Preferred Reporting Items for Systematic Reviews and Meta-Analyses (PRISMA) systematic evaluation guidelines [35]. PRISMA guidelines is a complete, rigorous, and credible tool, which are becoming more and more indispensable, especially in health-related fields [36]. A protocol was set in advance to improve the inclusion criteria and make the search for literature more comprehensive. Therefore, a search strategy was developed that can retrieve relevant studies with key terms appearing in the title, abstract, and keywords. Six databases were used for the literature search: (1) Web of Science; (2) Scopus; (3) ScienceDirect; (4) PubMed; (5) ProQuest; and (6) SportDiscus. These electronic databases encompass research related to exercise and health, which increases the likelihood that relevant studies will be retrieved and included. The Boolean logic operators were used in the electronic databases, with the search term "physical literacy" AND (teacher OR educator OR instructor OR schoolteacher OR lecturer OR coach OR mentor OR tutor). The term "physical literacy" is enclosed in double quotes to ensure that the search is for articles related to physical literacy, not "physical" or "literacy." Two systematic reviews [17, 37] that have had a profound impact on PL research guided the development of the protocol. The last search was conducted on April 3, 2023.

The criteria that can be included in this systematic review are (1) peer-reviewed publications, and (2) English publications. In order to meet the purpose of this study and address research questions, the exclusion criteria are as follows: (1) The research subject is not a physical education teacher; (2) The assessment method of PL (qualitative or quantitative) is not been reported; (3) book chapters and book reviews; (4) conference papers and readings; (5) theses or dissertations; (6) editorials and forewords; (7) not accessible in full text.

**Fig 1:**
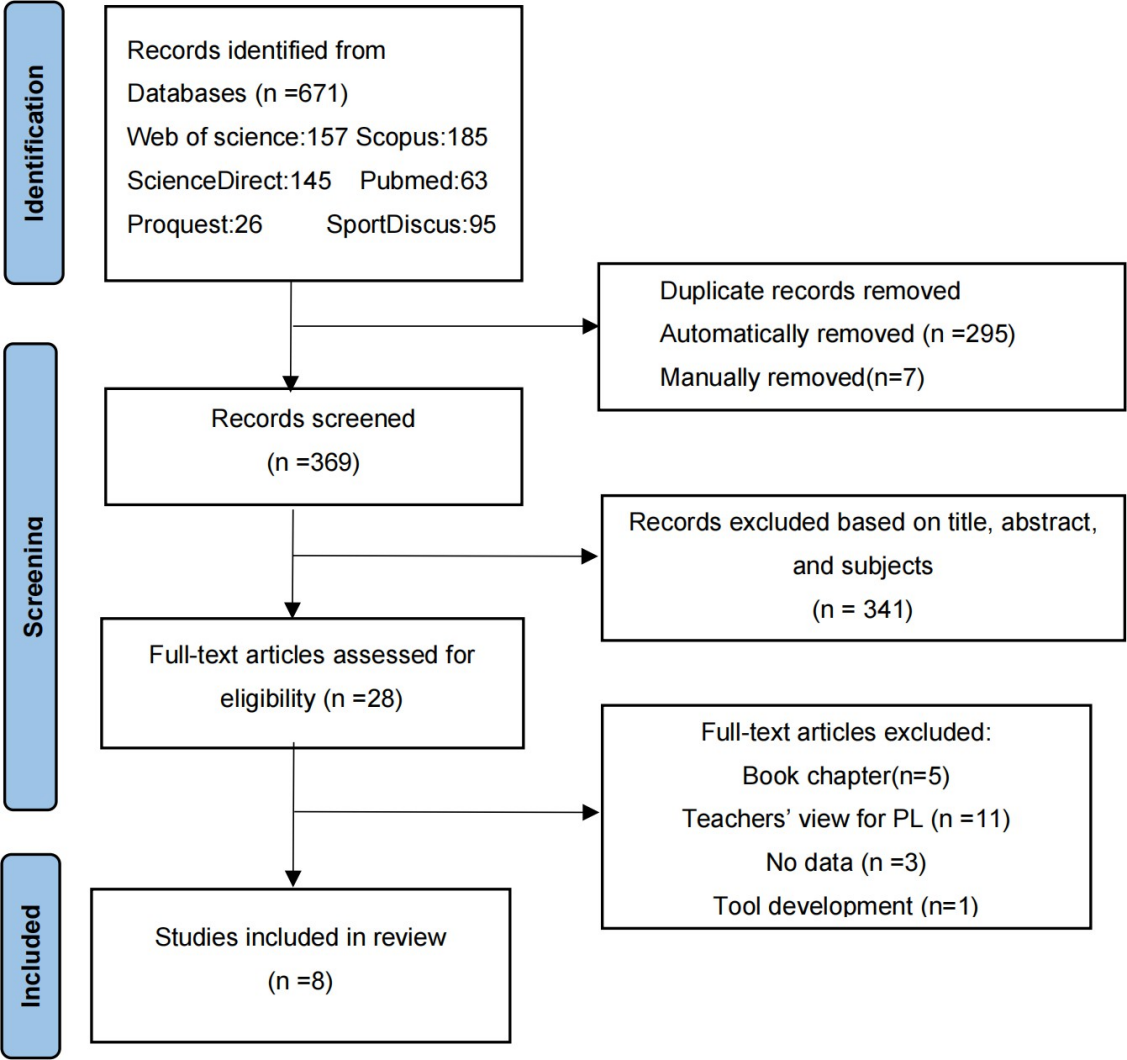
PRISMA flow diagram showing the process of study identification and selection [36].

The filtering results from all databases were imported into Excel software. After removing duplicate literature, two reviewers (YH and LF) independently conducted literature screening to evaluate whether these articles met the inclusion and exclusion criteria based on the title, abstract, and keywords. Subsequently, conduct a full-text review of the remaining articles. Any disputes or discrepancies between the two reviewers were resolved through discussion with the third reviewer (LM), and a consensus was reached that 8 articles met the criteria. Fig 1 shows the inclusion and exclusion of literature at each stage.

## 3 Results

### 3.1 Summary of studies

After identification and screening, only 8 articles out of 671 retrieved from the database finally met the preset eligibility criteria (for the detailed process, see Fig 1). Table 1 shows an overview of the included studies.

**Table 1:** Summary of studies.

Study	country/region	year	population (age; sex)	assessment purpose	assessment tool	assessment domain	assessment content	assessment form	assessment result
Chen [38]	USA	2022	Pre-service PE teachers (19–26; m = 23, f = 7)	Assessing PL compared to 12 years old children	Part of CAPL-1 and a cognitive questionnaire	physical cognitive	**Physical fitness:** body composition (BMI and waist circumference), musculoskeletal fitness (grip strength, plank), flexibility (sit and reach test), and aerobic capacity (20-m progressive aerobic cardiovascular endurance run, PACER). **Motor performance:** obstacle course (two-foot jumping, sliding, catching, throwing, skipping, one-foot hopping, and kicking). **Daily PA level:** wear research-grade accelerometers for the assessment of step counts. **Cognitive factors:**10 items questionnaire about physical activity, sedentary behavior, physical fitness, and safety during physical activity	physical measurement; device monitoring; online questionnaires	**Physical fitness:** flexibility (cm) (male = 31.51 ± 8.64, female = 39.99 ± 5.69) ^+^ grip (kg): (male = 99.15 ± 16.84) ^+^ BMI = 27.63 kg/m^2^ (overweight)^-^aerobic capacity (lap): (male = 33.78 ± 16.64, female = 19.29 ± 5.44)^-^plank test(lap):(M = 102.01 ± 16.64) ^-^ **Motor performance**: obstacle course^-^ (M = 23.93 ± 2.15) **Daily PA level** (step): (M = 14587.98 ± 5160.96) ^≈^ **Cognitive factors** (point): knowledge and understanding (M = 13.94 ± 2.02) ^+^
Judith [39]	Australia	2019	pre-service teachers from Bachelor of Education courses for Early Childhood and Primary (18–40 +; n = 57, sex = NR)	Assess PL to check the preparation of pre-service teachers	questionnaire (name unknown)	affective cognitive	**PPL**: APC; PLB; PLA **OPL:** SM; PTC; PLT	on-campus and online survey	**PPL (out of 5):** APC (Ave = 2.55); PLB (Ave = 3.11); PLA (Ave = 3.96) **OPL (out of 5):** SM (Ave = 3.57); PTC (Ave = 3.77); PLT (Ave = 2.18) The strongest areas are PLA, PTC, SM, and the weakest areas are APC PLT Total PL average = 3.19
Günay [40]	Turkey	2022	PE teacher (n = 161) Classroom teacher (n = 124) (NR; m = 113, f = 172)	Assess PL to compare the distinctions between PE teachers and classroom teachers	Turkish version of PPLI	affective cognitive	Sense of self and self-confidence; knowledge and understanding; self-expression and communication with others.	online and offline questionnaires	Sense of self and self-confidence (out of 5): M_PE_ = 4.67 ± 0.37, M_classroom_ = 3.81 ± 0.71 Knowledge and understanding (out of 5): M_PE_ = 4.77 ± 0.33, M_classroom_ = 3.82 ± 0.71 Self-expression and communication (out of 5): M_PE_ = 4.75 ± 0.31, M_classroom_ = 3.63 ± 0.72 Total PL (out of 5): M_PE_ = 4.72 ± 0.24, M_classroom_ = 3.81 ± 0.67
E. Jean [41]	Canada	2021	Early childhood educator (mean = 38; m = 5, f = 89)	Examine the PL of ECEs	OEE; BREQ-3; SEE; TGMD-2; PiezoRx pedometers	physical affective cognitive	Understanding the benefits of participation in regular PA; Motivation and confidence to Participate in exercise; Fundamental movement skills; One-week steps.	physical measurement; device monitoring; offline questionnaires	Motivation (range:-24-24): Mean = 11.5, SD = 6.4 Confidence (range:0–10): Mean = 5.0, SD = 1.9 Understanding (range:1–5): Mean = 4.2, SD = 0.6 PA (steps/day): (range:1000–40000) Mean = 11832, SD = 4744 PC (locomotor) (range:0–48): Mean = 39.0, SD = 4.0 PC (object control) (range:0–48): Mean = 36.7, SD = 6.2
E. Jean [42]	Canada	2021	Early childhood educator (19–51+; m = 1, f = 77, unknow = 7)	Examining the knowledge confidence of ECEs in PL and its application to practice	two questionnaires (name unknown)	affective cognitive	**cognitive:** PA knowledge; age of skill acquisition; error detection; error correction affective: confidence in some situations	online questionnaires	cognitive: M = 62.5% ± 16.6 affective: Mlocomotor skill (out of 10) = 6.4 ±1.7 Mmovement development (out of 10) = 7.3 ± 1.7
Choi [43]	Hongkong; Taiwan	2020	pre-service PE teachers (undergraduate; m = 126, f = 92)	Exploring the relationships and predictions concerning perceived PL and PE teaching efficacy.	PPLI; PETES	affective cognitive teaching efficacy	**PPLI:** Sense of self and self-confidence; knowledge and understanding; self-expression and communication with others. **PETES:** PE content knowledge; applying scientific knowledge in teaching PE; accommodating skill level differences; teaching students with special needs; instruction; using assessment using technology.	offline questionnaires	**PL score (out of 5):** knowledge and understanding = 4.50 ± .46 (highest) self-expression = 3.91 ± .60 (lowest) total PL = 4.18 ± 0.45 **TE score (out of 10):** instruction = 7.80 ± 1.12 (highest) teaching students with special needs = 5.56 ± 1.8 (lowest) total TE = 7.19 ± 1.07 The composite scores for perceived PL and PE TE (r = .49, p < .01), along with their attributes (r = .19 –.48, p < .01), were significantly positively correlated. For the composite score of both constructs, PE TE was predicted by perceived PL, F (7, 210) = 12.73, p < .001, R² = .30, significantly when controlling for the demographic variables.
Aaron [44]	Australia	2022	Primary school teacher (mean = 39.6; m = 16, f = 76)	Exploring the impact of OPD on the PL of elementary school teachers	interview; IMI; PETPAS; other instruments (name unknown)	cognitive affective	PL knowledge and application; PA facilitation; Value of PA; Value of PL; Confidence in planning and delivering a PL program; Need-supportive PA behavior; Perceived barriers to PA teaching	interview questionnaire	PL knowledge and application: Mbaseline = 7.26 ± 4.49, Mfollow-up = 7.30 ± 5.43 PA facilitation: Mbaseline = 3.24 ± 1.09, Mfollow-up = 3.33 ± .93 Value of PA: Mbaseline = 4.61 ± .43, Mfollow-up = 4.61 ± .47 Value of PL: Mbaseline = 3.95 ± .71, Mfollow-up = 3.89 ± .73 Confidence in PL programming: Mbaseline = 3.00 ± 1.00, Mfollow-up = 2.85 ± 1.10 Autonomy support: Mbaseline = 3.05 ± .76, Mfollow-up = 3.16 ± .60 Competence support: Mbaseline = 3.63 ± .79, Mfollow-up = 3.57 ± .73 Relatedness support: Mbaseline = 3.57 ± .87, Mfollow-up = 3.44 ± .88 Perceived organizational barriers: Mbaseline = 2.79 ± .92, Mfollow-up = 2.93 ± .98 Perceived personal barriers: Mbaseline = 2.40 ± .94, Mfollow-up = 2.61 ± .83
Chen [45]	USA	2022	Pre-service physical educators (19–25; m = 46, f = 14)	evaluate the PL of pre-service physical educators	Part of CAPL-2 PSPP	physical affective	**Height, weight, and Body composition:** BMI; Physical competence: CAMSA, plank test, PACER; **Physical activity level:** accelerometer and self-reported PA **Perceived physical competence:** perceptions of stamina and fitness, level of physical condition, confidence in an exercise and fitness setting, and ability to maintain exercise.	physical measurement; device monitoring; online questionnaire	**BMI** (kg/m2): male = 29.04 ± 5.40 (overweight)female = 24.74 ± 4.00 **Physical competence:** males = 24.17, t(45) = -5.671, p < .001(lower) females = 22.71, t(13) = -2.598, p = .022 (lower) **PA levels (steps/day):** males = 10587.31, t(41) = -5.44, P < .001 (fewer); females = 9916.39, t(12), p = .18 (fewer) **Perceived physical competence:** positive relationship between daily steps (r = .35, p = .009) and self-reported MVPA in a week (r = .354, p = .008)

USA United States of America, ^+^ better than children, ‐ weaker than children, ^≈^approximately equal to children, PL Physical Literacy, CAPL Canadian Assessment of Physical Literacy, PACER Progressive Aerobic Cardiovascular Endurance Run, PA Physical Activity, NR Not report, PPL Personal Physical Literacy, APC Actual Physical Literacy capability, PLB Physical Literacy Behaviors, PLA Physical Literacy Attitudes, OPL Teaching Attributes Organizational Physical Literacy, SM Teaching Mindset and Understanding of Physical Literacy Development for Children, PTC Personal Teaching Confidence, PLT Teaching Skills for Physical Literacy, ECE Early Childhood Educator, OEE Outcome Expectations for Exercise Scale, BREQ-3 Behavioral Regulation in Exercise Questionnaire version 3, SEE Self-Efficacy for Exercise Questionnaire, TGMD-2 Test of Gross Motor Development 2, PC Physical Competence, PPLI Perceived Physical Literacy Instrument, PETES Physical Education Teaching Efficacy Scale, OPD Online Professional Development, IMI intrinsic Motivation Inventory, CAPL-2 Canadian Assessment of Physical Literacy version 2, PSPP Physical Self-Perception Profile, CAMSA Canadian Agility, and Movement Skill Assessment

### 3.2 Characteristics of studies and participants

The characteristics of the included studies are shown in Tables 2 and 3. There are not many studies on the PL of PE teachers, and they are concentrated in developed countries or regions with better economic development, all of which have been conducted in recent years. The population mainly consists of PE teachers of children and adolescents and also includes pre-service teachers (individuals who are pursuing PE with the intention of becoming licensed teachers). The age of the population mainly ranges from young to middle-aged.

**Table 2:** Study characteristics.

Characteristics	Value
**Country or Region**	
USA	2
Australia	2
Canada	2
Turkey	1
Hongkong &Taiwan	1
**Year of Publication**	
2019	1
2020	1
2021	2
2022	4
**Objective**	
Pre-service teacher	4
Early childhood teacher	2
Primary school teacher	1
Middle and high school teacher	1
**Sample size**	
0–50	1
51–100	5
201–250	1
251–300	1

**Table 3:** Participants characteristics.

Characteristics	Value
**Gender**	
Male	330
Female	527
Unknown	64
**Age (Mean)**	
20–25	308
25–30	57
35–40	271
No report	285

### 3.3 Characteristics of assessment tools

Table 4 displays the assessment tools utilized in the studies incorporated in this review (tools whose names were not known were excluded). There were 12 instruments in total, including 7 developed in the United States, 4 developed in Canada, and 1 developed in Hong Kong. Among them, the Canadian Assessment of Physical Literacy (CAPL) measures four domains related to physical literacy: physical, cognitive, affective, and daily domain. The daily domain is measured through a pedometer and a self-reported questionnaire, which accounts for 32 points. The physical competence domain was measured by body composition (BMI and waist circumference), musculoskeletal fitness (grip, plank, flexibility), Progressive Aerobic Cardiovascular Endurance Run (PACER), and obstacle course, accounting for a total of 32 points. The cognitive and affective domains are both measured by questionnaires, each accounting for 18 points. The scores of the four domains are added together to obtain a total CAPL score (out of 100), and the total PL score is subsequently categorized into four levels: beginning, progressing, achieving, and excelling, depending on age and gender. In CAPL-2, the score for each domain has been changed to 30 points for the physical domain, 30 points for the daily domain, 30 points for the emotional domain, and 10 points for the cognitive domain. The physical competence domain is also simplified by removing the body composition test and retaining only the PACER, plank support, and CAMSA.

**Table 4:** Characteristics of the assessment instruments involved in the included studies.

Assessment tool, country/region of origin, author of primary study[citation]	Target population	purpose or use of assessment	Assessment domain	Constructs assessed	scale scoring	Validation	strengths	limitations
CAPL Canada HALO [46]	8–12 years old	Assess the PL of children	physical affective cognitive daily	Daily domain is assessed via self-report questionnaire and pedometer step counts; Physical domain is assessed via body composition (height, weight, wc), musculoskeletal fitness (grip, plank, flexibility), PACER, and obstacle course; Affective and cognitive domains are assessed via questionnaires.	The total score is 100 points, and the scores in the daily domain, physical domain, cognitive domain, and affective domain are 32 points, 32 points, 18 points, and 18 points, respectively. Children can be divided into four grades according to their total scores: beginning, progressing, achieving, and excelling.	The study has support that CAPL is valid (The Goodness of Fit Index was 0.96, and the Bentler Comparative Fit index was 0.94)	CAPL is a comprehensive assessment tool that broadly assesses all domains of PL	Time-consuming The assessor needs to undergo professional training
OEE USA Resnick [47]	older adults	Assessing what outcomes are expected from exercise participation in older adults	cognitive	13 items with two subscales: positive OEE and negative OEE	5-point Likert scale from 1 (strongly disagree) to 5 (strongly agree)	Good validity and reliability [47, 48]. (2001,2004)	suitable for older adults of different races	The activity was based on self-report, and research has indicated that self-report surveys frequently overestimate PL [49]
BREQ-3 Canada Wilson [50, 51]	general population	Assess an individual’s motivation to participate in exercise	affective	24 items to assess the 6 types of motivation in SDT	5-point Likert scale from 0 (not true for me) to 4 (very true for me)	A scale with good validity and reliability to assess motivation [50]	used broadly among researchers.	It is hard to translate in different languages and cultural contexts directly.
SEE USA Resnick [47]	older adults	Evaluate the confidence in one’s ability to self-regulate exercise even when encountering different obstacles or barrier	affective	9 items about exercise self-efficacy	10-point Likert scale	SEE is reliable, and it has good internal consistency [52].	Very reliable and valid.	Only suitable for older adults
TGMD-2 USA Ulrich [53]	3–10 years old children	Assess physical competence	physical	12 fundamental motor skills: run, gallop, hop, leap, horizontal jump, slide, strike, stationary dribble, catch, kick, overhand throw, and underhand roll	Each skill is assessed on three and five performance criteria.	TGMD-2 has good content validity, construct validity, and retest reliability [53, 54].	Convenient, with low requirements for venue equipment	The test indicators are selected from the cultural system of the United States, and some indicators have limited applicability.
PETES USA Humphries [55]	PE teachers	Assess PE teaching efficacy of PE teachers	cognitive	35 items scale with 7 subscales included "PE knowledge", "applying scientific knowledge in teaching PE," "accommodating skill level differences," "teaching students with special needs," "instruction," "using assessment," and "using technology."	10-point Likert scale from 1 (disagree/cannot do) to 10 (agree/highly certain I can do) with a midpoint of 5 (neutral/moderately certain I can do)	The study supports that PETES has good construct validity through confirmatory factor analysis [55].	A broader, multi-dimensional teaching efficacy instrument specific to personal teaching efficacy for PE	Multiple methods are required to verify validity and reliability.
PPLI Hongkong Sum [56]	PE teachers	Assess the perceived PL level of PE teachers	cognitive affective	9 items about motivation, confidence physical competence, and interaction with the environment.	5-point Likert scale	The study supports that PPLI has good construct validity through confirmatory factor analysis [56].	Further validated for youth and translated into multiple languages [57–60]	This study was based on self-reported perceived physical literacy so the results may be biased [56].
IMI USA Ryan [61]	general population	Assess a range of motivational constructs	affective	45 items with 7 subscales: interest/enjoyment, perceived competence, effort/importance, pressure/tension, perceived choice, value/usefulness, relatedness	5-point Likert scale from 1 (strongly disagree) to 5 (strongly agree).	Studies support that it has good validity and reliability.	Widely used worldwide [62–64].	It may not be closely related to PL
IBQ Canada Rocchi [65]	general population	assesses perceptions of interpersonal behaviors of others	cognitive	24 items with 6 subscales: AS, AT, CS, CT, RS, RT	7-point Likert scale from1 (do not agree at all) to 7(completely agree)	There were moderately high positive and negative correlations between the subscales.	widely used worldwide [66, 67]	It may not be closely related to PL
PETPAS USA Martin [68]	PE teachers	assess physical education teachers’ self-efficacy for teaching classes in which their students were engaged in high levels of physical activity	cognitive	16 items with 4 factors.	11-point Likert scale from 0% (not at all confident) to 100% (very confident)	It has good structural validity after exploratory factor analysis [68]	Good validity and high internal consistency.	The instrument only measures self-efficacy for promoting physical activity, which may not capture all aspects of a teacher’s self-efficacy or ability to teach PE
CAPL-2 Canada HALO [69–71]	8–12 years old	Assess PL of children	physical affective cognitive daily	Daily domain is assessed via self-report questionnaire and pedometer step counts; Physical domain is assessed via PACER, CAMSA, and plank. Affective and cognitive domains are assessed via questionnaires.	The total score is 100 points, and the scores in the daily domain, physical domain, cognitive domain, and affective domain are 30 points, 30 points, 10 points, and 30 points, respectively. Children can be divided into four grades according to their total scores: beginning, progressing, achieving, and excelling.	highly reliable and valid	The test is comprehensive and fits the definition of physical literacy, covering every dimension	Time-consuming; Assessors need to undergo professional training
PSPP USA [72]	college age	Assessment of self-perception in the physical domain.	perceived physical; affective	Five 6-item subscales to measure PSC, PBA, PPSM, PC, and PSW.	A four-choice structured alternative format was selected for item design.	Good validity was verified through exploratory factor analysis and confirmatory factor analysis.	The study reported good validity.	The results of self-reporting may be biased.

HALO The Health Active Living and Obesity Research, CAPL Canadian Assessment of Physical Literacy, PL Physical Literacy, OEE Outcome Expectations for Exercise Scale, USA United States of America, BREQ-3 Behavioral Regulation in Exercise Questionnaire version 3, SEE Self-Efficacy for Exercise Questionnaire, TGMD-2 Test of Gross Motor Development 2, PETES Physical Education Teaching Efficacy Scale, PPLI Perceived Physical Literacy Instrument, IMI intrinsic Motivation Inventory, IBQ Interpersonal Behaviors Questionnaire, PETPAS Physical Education Teacher Physical Activity Self-Efficacy Instrument, CAPL-2 Canadian Assessment of Physical Literacy version 2, PSPP Physical Self-Perception Profile

In addition to CAPL and CAPL-2, there is also a tool that measures the physical domain: the TGMD-2(Test of Gross Motor Development, Second Edition). The TGMD-2 is a set of tests comprising two subtests: namely locomotor and object control, each designed to assess distinct aspects of gross motor development. The locomotor subtest evaluates skills related to movement and coordination, while the object control subtest focuses on skills involving the control and manipulation of objects. The TGMD-2 takes a process score, with the examiner scoring the test based on the subject’s performance. In addition, PSPP measures the self-perceived physical domain. There are five subscales, each with 6 items. A four-choice structured alternative format was selected for item design. Each scale has a maximum score of 24 and a minimum score of 6. This includes perceived sports competence, perceived physical strength and muscle development, as well as perceived level of physical conditioning and exercise.

As for cognitive and affective domains, all tests are measured using online or offline questionnaires. All questionnaires are scored on the Likert scale rating system. It is important to note that while most questionnaires measure only one of the affective or cognitive domains, the Perceived Physical Literacy Instrument (PPLI) assesses both affective and cognitive domains, as well as a third domain, communicative domain, using nine questions to assess three domains of physical education teachers’ self-perceived physical literacy: the sense of self and self-confidence, self-expression and communication with others, and knowledge and understanding. Again, a Likert scale was used, with 1 indicating strongly disagree, 5 indicating strongly agree, and 3 indicating neither agree nor disagree.

### 3.4 Results of assessment

#### 3.4.1 Physical competence

There were three studies [38, 41, 45] that measured the physical competence of physical education teachers, in which Chen [38] compared the pre-service physical education teachers to that of 12-year-old students. The result showed that pre-service PE teachers were more flexible than 12-year-old students, but their aerobic capacity and motor performance were not as good as 12-year-old students, and their mean BMI was higher (27.63 kg/m^2^), indicating overweight status. Additionally, pre-service female PE teachers demonstrated lower levels of muscular strength and muscular endurance compared to 12-year-old students. Chen [45] assessed the PL of pre-service PE in another study and showed that self-assessed perceived physical competence (out of 24) was at a high level, with Mean (SD) = 18.13 ± 4.23 for males and Mean (SD) = 17.86 ± 6.10 for females. However, the actual physical competence results were quite different from the perceived physical competence. The BMI (29.04 kg/m^2^) indicated overweight status, and results for cardiorespiratory endurance, motor skill performance, and muscular endurance were all low. Buckler measured the physical competence of early childhood educators using the TGMD-2. The results showed that locomotor scores Mean (SD) = 39.0 (4.0) and object control scores Mean (SD) = 36.7 (6.2), with scores ranging from 0 to 48. Their physical competence is at a low level.

#### 3.4.2 Daily behavior or physical activity level

Chen’s [38] study found the daily activity level of pre-service PE teachers was M = 14,587.98 ± 5,160.96 (steps/day), which was not significantly different from that of 12-year-olds M = 15,000 (steps/day), t(29) = -.44, *p* = .667. The results of another study by Chen [45] showed that pre-service PE teachers’ daily physical activity levels were at a low level, with males = 10,587.31 (steps/day), t(41) = -5.44, *p*< .001 and females = 9916.39 (steps/day), t(12) = -2.745, *p* = .018. Buckler’s [41] study showed that early childhood teachers’ daily step mean (SD) = 11,832 (4744) (steps/day), which means that most participants have achieved the daily steps (8000–10000) required to stay healthy [73].

#### 3.4.3 Affective domain and cognitive domain

Seven studies assessed the affective domain, and essentially these studies showed that participants scored at the moderate or upper moderate level in the affective domain of PL. E. Jean’s study showed that early childhood teachers’ motivation Mean (SD) = 11.5 (6.4), with a score range of -24 to 24. Their confidence Mean (SD) = 5.0 (1.9), with a score range of 0 to 10. Judith’s study indicated that the score in the affective domain is Mean (SD) = 3.96 (0.59), which is the highest among all domains [41]. One study also assessed the affective domain scores of classroom teachers compared to PE teachers, M_PE_ = 4.67±0.37 and M_classroom_ = 3.81±0.71. A total of seven studies also evaluated the cognitive domain of PL of participants. The findings collectively indicated that the cognitive domain had the highest mean scores of all domains, regardless of whether they were pre-service or in-service teachers. For example, E. Jean found in one of his studies that early childhood teachers scored Mean(SD) = 4.2(0.6) in the cognitive domain, with a score range of 0 to 5. Participants scored highly on the general PA knowledge questions. However, there are difficulties in identifying information related to PA recommendations for early childhood (only 14% of participants answered accurately). This means that teachers have basic knowledge of physical activity, but they may struggle to effectively apply it to teaching methods or content for children [42].

## 4 Discussion

Physical literacy as a comprehensive and holistic concept has inspired scholars to explore and practice this concept globally. This systematic review aims to identify instruments/tools for measuring PL among PE teachers, review the level of PL demonstrated by PE teachers in existing studies, analyze underlying factors, and provide recommendations for future research. While most previous systematic reviews on PL have focused on adolescent children, older adults, or children with disabilities, this review uniquely focuses on PE teachers. As far as our knowledge extends, this is the first comprehensive systematic review of PL specifically among PE teachers, encompassing 8 included Studies that highlight limited exploration in this area.

In the 8 included studies, a total of 12 distinct quantitative assessments were then confirmed for validity and reliability. Among these, only three instruments (CAPL-1, CAPL-2, and PPLI) explicitly measured PL levels, with the remaining instruments assessing only one aspect of PL. The CAPL-1 was developed in 2014 by HALO (The Health Active Living and Obesity Research) in Canada. HALO organization, and considering the low relevance of the four dimensions of the CAPL-1 assessment [74], HALO revised it to promulgate the CAPL-2 in 2017, which not only streamlines the items but also optimizes the operational difficulty and time-consuming, making it one of the most popular and highly reliable physical literacy assessment tools today and it has been translated into several versions and is widely used worldwide [46, 75]. Although the CAPL is very popular in the PL assessment area, it is necessary to clarify that the CAPL is designed to be used with 8–12 years old children. In the two studies that reached the inclusion criteria [38, 45], the subjects were pre-service teachers, and the instrument used was the CAPL, which clearly does not fit the age range to which the CAPL is applicable. Hence, the scientific rigor and accuracy of the findings in these studies require further verification.

Another assessment tool that can systematically assess teachers’ PL levels is the PPLI (Perceived Physical Literacy Instrument), an instrument developed by Sum et al. in 2016 specifically to assess PE teachers’ perceived PL levels and was validated to demonstrate strong reliability and validity, including three dimensions: "sense of self-confidence," "self-expression and communication with others," and "knowledge and understanding" [56]. The PPLI was further validated to be equally applicable to adolescents and older adults, with good reliability and validity [57, 76]. Then the PPLI has since been translated into multiple language versions for use in various countries [58–60]. However, one study has shown that PL’s behavioral, psychological, and physical competence is (theoretically and practically) distinct but interrelated and that a comprehensive assessment of the constructs can offer a more precise assessment of a person’s ability to perform PL. To fulfill the purpose of PL assessment, a suitable evaluation of physical skills is essential. [77], which aligns with the concept of PL defined by Whitehead [18]. Furthermore, it has been established that there is a difference between children’s perceived PL and actual PL [78]. In addition, it has also been noted that self-report-based tests of physical competence are not credible [79], so many researchers recommend the use of objective direct measures of physical competence [80]. Therefore, whether there is a difference between the perceived PL of PE teachers, as measured by the PPLI, and the actual PL of teachers needs to be confirmed in future studies.

Other PL assessment tools assess only one domain of PL. In other words, these assessment tools themselves were not developed to assess a person’s PL, only that what they assess happens to align with one or more domains of the PL definition, enabling indirect assessment of certain aspects of PL. For example, physical competence constitutes a crucial component of PL, and within the concept of PL, there is an overlap in meaning between physical competence and some of the terms commonly used in more established fields of current research, such as motor ability, motor control, motor skills, etc. [81–83], and the TGMD-2 included in this review assesses an individual’s motor skills. Meanwhile, due to the relative paucity of tools to assess PL, some researchers have used these tools to assess PL. Although researchers used assessment tools to measure the cognitive and affective domains in the included studies, the lack of a gold standard led to mixed results across assessments. This is because many different factors are included in the affective and cognitive domains. For example, the affective domain includes motivation, confidence, enjoyment, commitment, autonomy, and self-esteem; the cognitive domain includes the knowledge and understanding of the benefits of PA, the knowledge and understanding of the importance of PA, knowledge, and understanding of strategies, rules, and assessment of safety considerations and risks [84]. Moreover, the tools used in the included studies did not assess applied physical competence in different contexts or specific contextual knowledge focused focus on PA (e.g., strategies and organization) which are critical for PE teachers and align more closely with the definition of PL [85]. There are, of course, some valuable assessment tools. The BREQ-3 [50], for example, was developed using self-determination theory, widely recognized as a fundamental framework for comprehending motivation, not only in the context of sports but also more broadly in exercise and PA [85, 86].

This review also reviewed the PE teachers’ PL level in existing studies. Physical competence and level of daily PA were the areas of PL in which PE teachers (including pre-service PE teachers) performed poorly. Three studies [29, 32, 36] measured the physical competence of PE, and all three showed low levels of physical competence. Chen’s [38] findings indicate that pre-service PE teachers’ physical competence was, in some areas, inferior to that of a 12-year-old child. It is difficult for a PE teacher to serve as a teacher role model and for the PE teacher’s authority to be challenged if he or she is still inferior to a child as a responder to school PE policy, an implementer of PE, and a role model for children. In addition, given the unique nature of the role of PE teachers relative to teachers of other subjects, poor physical competence will directly affect the effectiveness of PE teaching as they are required to demonstrate some movements in the PE classes [18, 87, 88]. The reasons for the poor physical competence of PE teachers are multiple, including work ability [89], stress level [90], and lack of PE teacher development training [91], etc. In addition, aging is also a major factor affecting the physical competence of PE teachers, which can lead to the turnover or attrition of PE teachers [92]. Aging brings about a decline in physical competence and this process is irreversible. Nevertheless, PE teachers can exercise regularly to slow down this process [93, 94]. Conversely, PE teachers performed better in cognitive and affective than physical competence. The included studies showed moderate or high performance levels in the cognitive, and affective domains. This may be due to relevant courses taken during school years, current teacher development training, and accumulation of teaching work experience [44]. However, it should be noted that this study also takes pre-service teachers into account due to the limited number of included studies. The definition of pre-service teachers may vary from country to country, but they are still in the training stage and have not obtained a license or entered the workplace. Therefore, they are different from in-service teachers. The results of the included studies also show that there are differences between in-service teachers and pre-service teachers. Nonetheless, the results of this study are still applicable. Including pre-service teachers in the study can expand the research perspective, more comprehensively reflect the current situation and needs in the field of education, and better respond to future educational challenges.

Different groups have different feelings about PL and the content of PL. For PE teachers, the physical competence domain of PL includes their own athletic ability and physical health level that can support them in PE teaching activities, which is the foundation for a PE teacher. The affective domain includes the motivation and confidence of PE teachers to participate in PA and teaching activities, as well as the evaluation of their own and students’ various skills and abilities in PA and PE teaching activities, and communication and interaction with students. The perfect cooperation between PE teachers and students can lead to an excellent PE class. The cognitive domain includes PE teachers’ knowledge and understanding of PA and PE teaching activities, the ability to make informed decisions in the face of various emergencies, and how to adapt themselves and their students to various environments.

As mentioned before, the level of individual PL supports teaching ability in this area [39], and the level of PL also predicts the effectiveness of PE teaching [43]. Therefore, improving and maintaining a high level of PL among PE teachers is also a top priority in school PE. This is an issue that requires significant attention and action from educational policymakers. The teacher’s continuing professional development (CPD) is considered an effective way to improve the PL level of PE teachers. UNESCO’s guidelines for policymakers state that the CPD of teachers should be a priority for developing quality physical education(QPE) in each country [95]. There are already many countries where the CPD of teachers is a mandatory requirement for upholding teacher registration or maintaining teaching standards [96–98]. It has been established that the impact of teachers’ professional development on PE teachers’ PL is significant [44]. However, the content of teacher professional development programs will vary from country to country, with different cultural contexts and realities. Nevertheless, continuous professional development for teachers requires 1) attention to the complexity of the learning process, 2) prioritizing the context of the times and contemporary challenges, 3) integrating research/theory and practice in an innovative way, and 4) fostering the professional development of PE teachers [99]. Based on this, Sum et al. [100] developed PE-CPD specifically for PE teachers in Hong Kong by incorporating the concept of PL in the CPD. The study’s findings demonstrated a significant impact of customized PE-CPD on PE teachers’ beliefs concerning both perceived PL and teaching efficacy. The weakness is that there is a difference between actual PL and perceived PL. Also, with the rapid development of technology in the field of education, more CPD is now conducted in an online format [101, 102]. While online CPD can effectively improve physical literacy’s affective and cognitive domains for PE teachers, it is minimally helpful in their weakest domain, physical competence [103–105]. PL can be developed through PA, so it is necessary to include PA in CPD. Introducing PA as content to PE teachers also means that they can engage in the pursuit of PE [40]. Therefore, when developing CPD for PE teachers in the future, relevant policymakers should consider incorporating the relevant construct of PL, focusing on the overall development of PE teachers’ PL, selecting appropriate training content (e.g., sports with local characteristics, PE teaching methods that incorporate the concept of PL, knowledge of relevant content or relevant scenarios, etc.), and selecting appropriate training formats. (e.g., seminars, workshops, courses, etc.).

Overall, based on the included studies, our findings indicate that there is very little current research on PE teachers’ PL; only one instrument is available to assess PE teachers’ perceived PL, and there is no instrument available to assess PE teachers’ actual PL comprehensively. Moreover, the cognitive and affective of PE teachers are at moderate or high levels, and their physical competence is at a poor level. Future research on this topic could focus on developing tools that can be used to assess the actual PL of PE teachers systematically and comprehensively and how to improve the PL of PE teachers. In developing assessment tools, it is crucial to first unify the concept of PL within the PE teacher population, as the diversity of PL definitions can lead to confusion in PL assessment. Due to different definitions and philosophical underpinnings of PL, the components and methods of PL assessment are different [17, 77]. Indeed, the definition of PL is still a controversial topic [106], and the most widely accepted definition of PL is that of Whitehead [18], on which most PL assessment tools for children have been developed. However, the applicability of this definition to the PE teacher population, or the need to add additional elements, is a topic for future research. Secondly, the traditional/conventional, linear approach no longer meets the current needs of PL assessment [37], but rather an innovative, non-traditional approach should be used. For example, some argue that the results of evaluating PL should not be an intuitive reflection of the concept but rather suggest "charting" the progression of PL [107], which may be a normative and appropriate assessment method. Finally, existing assessment tools are deficient, and future tool development should take them into account. Ensuring that the assessment process is efficient and economical (human and material resources) and that the assessment results are scientific, valid, and accurate is essential. The CPD is an effective way to improve PE teachers’ PL. Future research on the CPD should consider the actual national context, cultural characteristics, governance structures, and the training courses’ novelty and challenge to increase PE teachers’ willingness to participate. Focus on the choice of training content and format to ensure that PE teachers who have participated in the CPD can effectively improve PL and can apply it to their daily PE teaching to enhance students’ PL.

This study has the following limitations: (1) Language bias: only papers published in English were retrieved. Therefore, studies on PL levels of PE teachers in non-English speaking countries may have been missed. (2) All retrieved articles were already published; therefore, some studies, such as the gray literature, may have been missed. (3) no qualitative assessments of physical education teachers’ physical literacy were found in the search results. If some specific qualitative methods (e.g., interviews, focus groups, etc.) were included in the search terms, the search results might contain some qualitative assessments. (4) The systematic review did not answer or present a concept about the PL of PE teachers, which may be considered a limitation.

## 5 Conclusion and implications

This study represents the first comprehensive paper that offers a systematic review of empirical research related to assessing or measuring PL among PE teachers. The current findings suggest that PE teachers perform poorly in the physical competence domain, and perform better in the cognitive and affective domains. However, there is insufficient literature to analyze the trend of PL among PE teachers.

Nonetheless, this study has important practical application value. First, identifying the deficiencies in the PL level of PE teachers can provide key data support for education departments and policymakers to help them formulate more targeted training and development plans. In particular, CPD programs are considered to be an effective means to improve PE teachers’ PL. By designing and implementing specialized CPD programs, the physical competence and overall PL level of PE teachers can be effectively improved.

Furthermore, there is currently only one assessment tool that can systematically measure PE teachers’ perceived PL (not actual PL); other studies have used instrumental components. This shows that there is an urgent need to develop more comprehensive and systematic assessment tools. These tools can not only help teachers self-assess and improve but also provide researchers with more reliable data to further promote the development of research and practice in the field of PL.

Therefore, future research should first attempt to define a unified concept of PL applicable to PE teachers and then develop tools that can comprehensively assess PL of PE teachers based on this concept. Furthermore, given the small number of studies that could be included at present, future research needs to expand the study criteria to include a wider population, such as teachers or general adults. This would provide a richer database, offer more reliable insights, and aid in the development of comprehensive assessment tools. Tool developers may consider combining qualitative or quantitative approaches in the future. In summary, the findings of this study provide an important reference for the future development of PL assessment tools and training programs for PE teachers, which will help improve the quality of PE and ultimately promote students’ participation in PA and the development of PL levels.

## Supporting information

S1 Checklist PRISMA 2020 checklist. (DOCX)
